# Unraveling the Scientific Landscape of Osteoarthritis: Dynamics of Publications over Five Decades

**DOI:** 10.3390/bioengineering12060602

**Published:** 2025-05-31

**Authors:** Roxana Maria Sanziana Pavel, Andrei-Flavius Radu, Ada Radu, Bogdan Uivaraseanu, Gabriela Bungau, Delia Mirela Tit, Delia Carmen Nistor Cseppento, Paul Andrei Negru

**Affiliations:** 1 Doctoral School of Biological and Biomedical Sciences, University of Oradea, 410087 Oradea, Romania; pavel.mariaroxanasanziana@student.uoradea.ro (R.M.S.P.); gbungau@uoradea.ro (G.B.); dtit@uoradea.ro (D.M.T.); dcseppento@uoradea.ro (D.C.N.C.); negru.paulandrei@student.uoradea.ro (P.A.N.); 2 Department of Psycho-Neurosciences and Recovery, Faculty of Medicine and Pharmacy, University of Oradea, 410073 Oradea, Romania; 3 Department of Pharmacy, Faculty of Medicine and Pharmacy, University of Oradea, 410028 Oradea, Romania; 4 Department of Surgical Disciplines, Faculty of Medicine and Pharmacy, University of Oradea, 410073 Oradea, Romania; buivaraseanu@uoradea.ro; 5 Department of Preclinical Disciplines, Faculty of Medicine and Pharmacy, University of Oradea, 410073 Oradea, Romania

**Keywords:** osteoarthritis, WOMAC, KOOS, bibliometric study, Python, VOSviewer, clinimetric tools, psychometric tools

## Abstract

Osteoarthritis is a disabling condition with highly complex overall management and persistent shortcomings, contributing significantly to the global disease burden. Although research in the field has grown considerably in recent years alongside technological advancements, a cohesive and structured understanding of the evolution of the scientific literature, particularly regarding clinical management and outcome evaluation, remains insufficiently developed. To date, most bibliometric analyses in osteoarthritis have focused narrowly on specific subdomains, leaving a notable gap in comprehensive assessments of the broader clinical framework. This study addresses that gap through an integrated, structured, and visual approach using multiple bibliometric techniques targeting osteoarthritis diagnosis and management, aiming to guide future research and improve strategic development. Scientific publication in osteoarthritis has expanded exponentially, peaking in 2024 with 1234 documents. The United States led in both output and citation impact, while China showed rapid growth. *Osteoarthritis and Cartilage* emerged as the most influential journal. Australian institutions, especially the University of Sydney, demonstrated a remarkable ascent. Five global research clusters were identified, with the U.S. as the central node and Australia serving as a bridge between Western and Asian collaborations. Research themes evolved toward integrated models connecting biological mechanisms, therapeutic strategies, and patient-centered outcomes. This bibliometric assessment underscores exponential growth in osteoarthritis research and highlights the urgent need for more personalized, multidimensional evaluation strategies to enhance clinical translation.

## 1. Introduction

The global rise in osteoarthritis incidence can be primarily attributed to demographic shifts, particularly an aging population, along with the escalating rates of obesity. This condition, recognized for its heterogeneity, poses a significant public health challenge due to its growing prevalence and associated disability. Recent research has deepened understanding of osteoarthritis, highlighting genetic factors, gut microbiota, and varied pain mechanisms as key contributors [1]. The anatomical regions most frequently implicated in osteoarthritis are the load-bearing joints, particularly the knee, hip, and lumbar spine, while the hand serves as a representative example of a non-load-bearing joint that is also commonly affected [2].

The etiology of osteoarthritis is multifactorial, involving diverse biological and mechanical contributors. Its complex pathogenesis is driven by a combination of mechanical, genetic, metabolic, inflammatory, and aging-related factors. Mechanical stress, often due to abnormal joint loading, malalignment, or injury, contributes to cartilage breakdown by inducing chondrocyte apoptosis and extracellular matrix degradation [3]. Genetic susceptibility plays a critical role in osteoarthritis development, with multiple gene variants influencing cartilage homeostasis and inflammatory pathways, explaining interindividual variability in disease onset and progression [4]. Osteoarthritis has been categorized into six primary phenotypes, each reflecting distinct pathophysiological pathways, including a chronic pain phenotype involving central sensitization, an inflammatory phenotype, a form associated with metabolic syndrome, a subtype characterized by altered bone and cartilage metabolism, a mechanically driven phenotype related to joint malalignment, and a phenotype with minimal joint structural changes, often presenting with milder clinical features [5].

Metabolic syndrome components such as obesity, insulin resistance, and dyslipidemia further exacerbate osteoarthritis through systemic low-grade inflammation and altered adipokine secretion, which negatively impact cartilage metabolism and promote catabolic signaling within the joint [6]. Inflammation, previously considered secondary, is now recognized as a key driver of osteoarthritis pathology. Pro-inflammatory cytokines like interleukin-1 beta and tumor necrosis factor-alpha contribute to sustained synovial inflammation, cartilage matrix breakdown, and pain sensitization [7]. Aging itself impairs chondrocyte function and decreases the regenerative capacity of cartilage and subchondral bone, facilitating progression in older individuals through oxidative stress and mitochondrial dysfunction [8]. This comprehensive understanding of osteoarthritis etiology thus provides essential context for selecting targeted interventions such as anti-inflammatory injections, regenerative therapies, or physical rehabilitation.

In 2021, osteoarthritis represented a substantial global health concern, with an estimated 606.5 million individuals living with the condition, and approximately 46.6 million new cases were recorded, contributing significantly to the overall disease burden. Furthermore, the disorder was responsible for around 21.3 million years lived with disability, underscoring its impact on quality of life and functional capacity. When considering incidence, the distribution diverges from that of prevalence, with the highest rates observed in the 50–54 age bracket. After this peak, a gradual decline is noted, reaching its lowest point in individuals aged 90–94 [9]. Projections indicate that by 2040, the prevalence of arthritis among adults in the United States is expected to reach approximately 78 million, representing a substantial increase in disease burden over the coming decades [2].

Osteoarthritis involves the breakdown of the cartilage in joints and the structural components of the bone matrix. Early changes predominantly affect type II collagen and the proteoglycan aggrecan, resulting in the deterioration of both the integrity and functionality of the cartilage [10].

Management of osteoarthritis involves several key approaches, including physical activity, patient education, and weight control when indicated. In cases of significant joint damage and advanced symptoms, total joint replacement surgery may be necessary. Nonsteroidal anti-inflammatory drugs, administered either orally or topically, are often prescribed for pain relief in individuals without contraindications [11]. Additionally, intra-articular steroid injections can provide short-term pain relief. Recent studies have highlighted the potential of compounds like cathepsin K inhibitors, Wnt pathway blockers, and anabolic growth factors to slow the structural progression of osteoarthritis, while nerve growth factor inhibitors may help reduce pain. Opioid use, however, should be avoided due to its potential risks [12,13].

Managing osteoarthritis effectively involves both clinimetric and psychometric evaluations. The use of clinimetric tools allows for the assessment of clinical outcomes, providing insights into the severity of symptoms, functional restrictions, and the overall effect on the individual’s quality of life [14].

Commonly used clinimetric tools include the Western Ontario and McMaster Universities Osteoarthritis Index (WOMAC) and the Knee Injury and Osteoarthritis Outcome Score (KOOS), which allow for the systematic evaluation of pain, stiffness, and physical function in patients with OA [14,15]. Originally derived from the WOMAC, the KOOS was designed to evaluate the symptoms and functionality of individuals with knee injuries and osteoarthritis over both short and long durations. It includes five separate subscales, with each subscale assigned an independent score, each focusing on a different aspect: pain, function in sports and recreation, knee-related quality of life, function in daily living, and other symptoms [16].

The psychological impact of osteoarthritis is an important consideration, with chronic pain and disability often leading to increased anxiety, depression, and diminished coping abilities. To assess these psychological dimensions, psychometric tools complement clinimetric evaluations. Scales such as the Hospital Anxiety and Depression Scale and the Pain Catastrophizing Scale are useful for identifying patients who might benefit from psychological interventions or rehabilitation programs focused on enhancing mental health outcomes [17].

Bibliometric analyses play a vital role in advancing scientific knowledge and assessing the state of research. These analyses offer significant advantages, including the identification of research gaps, the definition of future research directions, and the mapping of influential authors, institutions, collaborations, and high-impact journals. In the field of osteoarthritis, several bibliometric studies have already made important contributions, focusing on targeted areas such as foot osteoarthritis [18], signaling pathways [19], or extracellular vesicles [20], etc. However, despite these valuable insights, there remains a notable gap in the literature when it comes to comprehensive bibliometric evaluations that synthesize research across broader dimensions of osteoarthritis, particularly in relation to general clinical management strategies and measurement tools. This gap highlights the need to build upon and contextualize existing findings by offering a more integrative overview that captures research dynamics across the wider clinical and methodological spectrum of osteoarthritis care.

The aim of this bibliometric assessment is to provide a comprehensive, quantitative, visual, and distinct assessment of the scientific literature dedicated to the diagnosis and management of osteoarthritis. Emerging trends, collaborative networks, influential journals and institutions, and existing research gaps for guiding future efforts in a more integrated and efficient way were identified. Therefore, this paper offers an original contribution, providing a systematic framework for understanding scientific progress from a holistic perspective and helping to define relevant future directions for applied research and clinical practice in osteoarthritis.

## 2. Materials and Methods

The primary data source for this bibliometric analysis was the Web of Science Core Collection. The selection of Web of Science Core Collection as our sole bibliographic database was an intentional methodological choice based on careful consideration of the recent comparative database literature and our specific research objectives. While multi-database approaches might theoretically enhance coverage, recent comprehensive evaluations demonstrate significant methodological challenges with such approaches [21]. WOS was selected based on several methodological considerations. First, WOS provides comprehensive bibliographic records with minimal missing abstracts, thereby enabling the robust text analysis required for keyword co-occurrence mapping and thematic evolution tracking. Second, the database ensures extensive coverage of multidisciplinary literature pertinent to osteoarthritis research, encompassing publications across medical, rheumatological, orthopedic, and rehabilitation domains. Finally, the utilization of a single, comprehensive database circumvented the methodological complexities inherent in cross-database integration, thus enhancing the reliability and reproducibility of the analytical framework.

Its selection was based on its extensive coverage of high-quality scientific literature and robust indexing system, which facilitate accurate bibliometric evaluations. A refined search algorithm was created using field tags and Boolean operators to ensure comprehensive retrieval of all pertinent studies on the diagnosis and management of osteoarthritis.

The specific formulation of the search query is as follows: ALL = (osteoarthriti* OR “degenerative joint disease” OR “OA”) AND ALL = (“clinimetric*” OR “psychometric*” OR “measurement properties” OR “validity” OR “reliability” OR “responsiveness” OR “outcome assessment” OR “WOMAC” OR “KOOS” OR “quality of life” OR “QoL” OR “pain assessment” OR “functional disability” OR “postural control” OR “balance assessment” OR “psychological impact” OR “anxiety” OR “depression” OR “treatment adherence” OR “personalized interventions”) AND ALL = (“hip osteoarthritis” OR “knee osteoarthritis” OR “lower limb osteoarthritis” OR “joint pain” OR “cartilage degradation” OR “joint stiffness” OR “physical function” OR “rehabilitation” OR “total knee replacement” OR “total hip replacement” OR “non-surgical treatment” OR “surgical outcomes” OR “exercise therapy” OR “NSAIDs” OR “opioids” OR “hyaluronic acid” OR “corticosteroids” OR “mesenchymal stem cells” OR “platelet-rich plasma”).

The Boolean operator ‘AND’ was used to ensure that retrieved documents included all three thematic categories, thereby increasing the specificity of the search results. Within each category, the ‘OR’ operator incorporated synonyms and related terms to broaden the search scope within specific conceptual boundaries. The asterisk (*) served as a truncation symbol to capture various word forms, such as ‘osteoarthritis’ and ‘osteoarthritic.’ Quotation marks enclosed exact phrases to prevent false positives caused by disconnected or unrelated term occurrences.

The initial search retrieved 15,502 documents. We subsequently refined this dataset to improve its suitability for bibliometric analysis by applying two key filters aimed at enhancing homogeneity and relevance. First, we retained only publications classified as ‘articles’ by Web of Science, excluding other formats like reviews and proceedings to minimize methodological heterogeneity. Second, we included only English-language articles to maintain consistency in our analysis and because most of the high-impact osteoarthritis research is published in English. This language restriction was particularly important for our keyword co-occurrence and thematic analyses, as including non-English publications would introduce terminological heterogeneity that could skew the weighting of concepts. For instance, keywords from non-English articles would appear with lower frequencies compared to their English equivalents, potentially underrepresenting important themes despite their actual prevalence in the global literature. Additionally, automated keyword standardization and clustering techniques are more reliable when applied to a single language, ensuring more accurate identification of research trends and thematic evolution. After applying these filters, our final dataset contained 12,661 articles.

The bibliometric analysis employed several software tools. Network visualizations such as co-authorship, co-citation, and keyword co-occurrence maps, which illustrate collaboration patterns and thematic clusters were created using VOSviewer (version 1.6.20). Statistical analyses and trend evaluations were conducted with the Bibliometrix 5.0.0 package within the R environment, accessed via the Biblioshiny interface, to calculate performance metrics at various levels including countries, institutions, journals, and authors. Additionally, Microsoft Excel facilitated data processing, tabulation, and visualization to enhance interpretability [22,23,24].

For the visualization of scientific networks, specific parameters were established to ensure clarity and relevance of the generated maps. In the country collaboration network, all nations with a minimum of 30 published articles were included in the analysis. The node size in this visualization corresponds to the volume of publications attributed to each country, with larger nodes representing more prolific nations. Lines connecting nodes represent collaborative relationships, with line thickness proportional to the intensity of collaboration between the connected countries.

For keyword co-occurrence analysis, terms appearing in at least 300 articles were included in the network visualization. The node size in this map reflects the frequency of occurrence, with more commonly used terms depicted as larger nodes. The proximity of nodes corresponds to the frequency of co-occurrence, while node colors designate cluster membership based on thematic relatedness determined by the VOS clustering algorithm.

Thematic evolution maps were created to show the chronological progression of research themes in osteoarthritis assessment and treatment. These maps were constructed using Bibliometrix’s co-word analysis and clustering techniques, and they divide the 50-year study period (1975–2025) into three separate stages depending on publication volume. This method allows for the detection of emerging, decreasing, and permanent themes, as well as the visualization of conceptual shifts over time.

**Figure 1:**
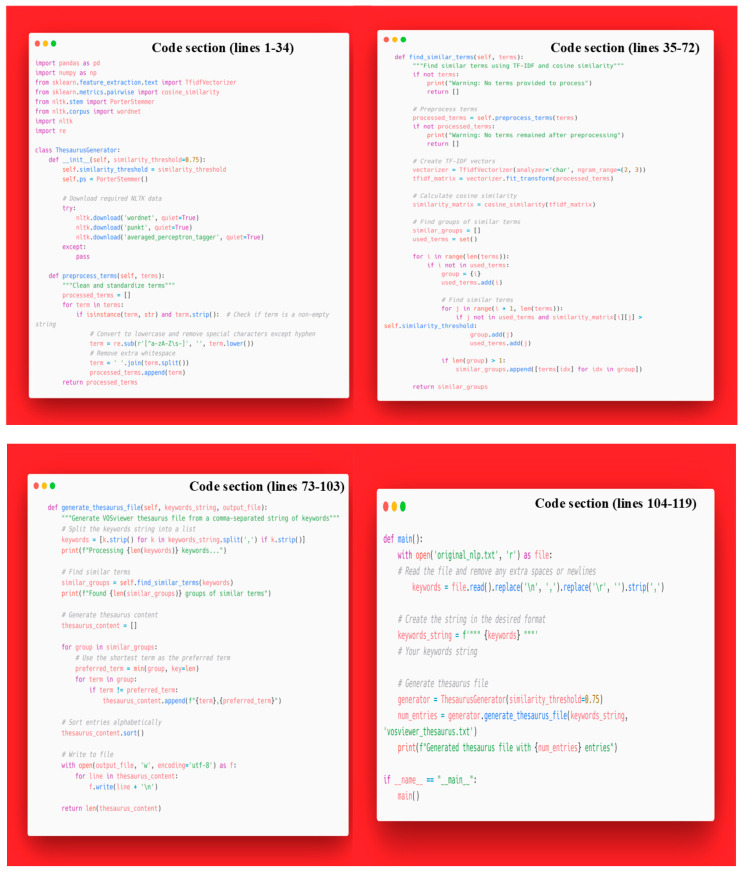
Custom Python script for keyword normalization.

To enhance the precision of keyword analysis, a standardization process was implemented to address variations in terminology. This process involved the harmonization of synonymous terms, the reconciliation of singular and plural forms, and the consolidation of abbreviations with their full forms. While country names and institutional affiliations were manually standardized, the large volume of keywords necessitated an automated approach. A custom thesaurus generator was developed using Python 3.12.3 to identify and cluster semantically related terms, employing character-level Term Frequency–Inverse Document Frequency (TF-IDF) vectorization with n-grams and cosine similarity metrics. This process designated the shortest term in each cluster as the canonical representation, resulting in a standardized lexicon for keyword analysis (Figure 1).

## 3. Results

### 3.1. Literature Overview

**Figure 2:**
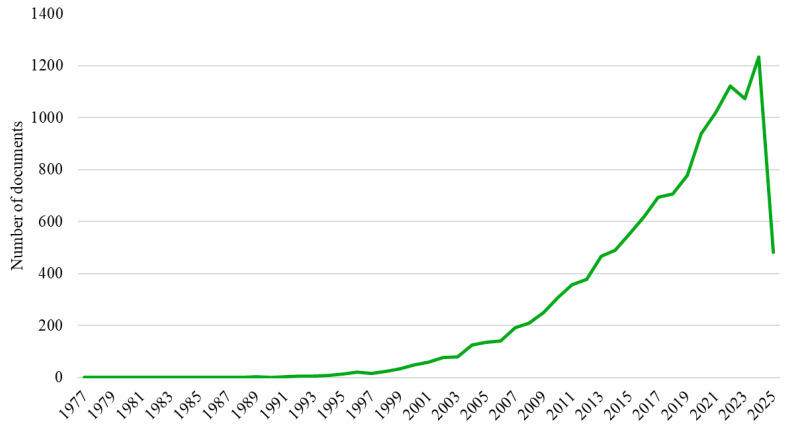
Temporal distribution of scientific publications.

The analyzed Web of Science dataset encompasses publications from 1977 to early 2025. Our analysis of publication trends reveals four distinct phases of research productivity (Figure 2). An initial ‘emergence’ phase (1977–1994) was characterized by minimal scientific output, rarely exceeding four documents annually. This was followed by a ‘gradual growth’ period (1995–2009), during which annual publications rose substantially from 14 (in 1995) to 249, establishing the field. Subsequently, a phase of ‘marked acceleration’ (2010–2019) saw output climb from 309 to 778 publications per year, coinciding with increased recognition of osteoarthritis as a global health issue and advancements in measurement and therapeutic strategies. The most recent ‘expansion’ phase (2020–2024) demonstrated unprecedented growth, exceeding 1000 annual publications and peaking at 1234 documents in 2024. Although the data for 2025 are incomplete, they suggest continued robust research activity. As depicted in Figure 2, the overall publication trajectory shows exponential growth, particularly over the last decade, reflecting significantly intensified research focus on osteoarthritis assessment and treatment.

**Figure 3:**
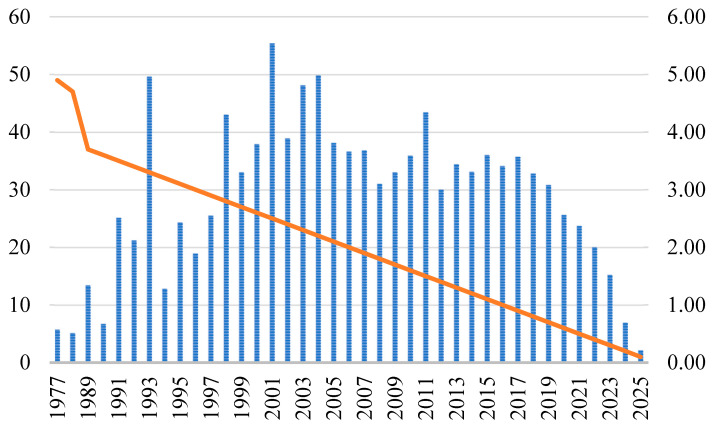
Temporal trends in for osteoarthritis assessment and treatment research (1977–2025). Data for 1988 (MeanTCperYear = 167.92) were excluded to improve visualization.

Temporal trends in citation impact were analyzed using Mean Total Citations per Year (MeanTCperYear), visualized in Figure 3. Impact varied considerably over time. Publications from 1977 to 1990 generally had low MeanTCperYear values (<1.0), with the notable exception of 1988 (167.92, due to a highly cited foundational work). Citation impact increased between 1991 and 2004, reaching a maximum of 5.54 in 2001, coinciding with the consolidation of assessment and treatment standards. The research field showed signs of maturation, as evidenced by the MeanTCperYear values, which stabilized between 2005 and 2019, oscillating typically between 3.0 and 4.0, with a peak of 4.34 in 2011. The observed decline from 2020 (2.56) to early 2025 (0.21) is primarily due to citation lag, the time delay before new papers accumulate citations, rather than a decline in research quality. These recent values are expected to increase in the future. Despite a substantial increase in publication volume, the average citation rate per paper has remained relatively steady over the past two decades, as shown in Figure 3. This indicates that the average influence of individual studies has not significantly changed despite the exponential growth in research output.

### 3.2. Global Scientific Productivity

Analysis of the 125 contributing countries (1977–early 2025) reveals leadership patterns in osteoarthritis assessment and treatment research (Top 10 in Table 1). The United States leads across all key metrics: publications (3094), citation impact (39.80/doc), and international collaboration (Total Link Strength, TLS = 2000). China is second in productivity (1080 articles) but lags substantially in citation impact (12.70/doc). The UK (1045 articles, 38.27/doc) and Australia (941 articles, 39.04/doc) show balanced high output and impact. Canada (841 articles) stands out for its exceptional citation impact (55.89/doc), the highest in the top 10, suggesting highly influential research. European nations feature prominently, with Denmark (474 articles, 42.20/doc) also showing high impact, alongside the Netherlands, Germany, and Italy maintaining strong impact scores (31–42/doc). Japan (16.43/doc, TLS = 159) joins China as the main Asian contributors, though both show lower impact than leading Western nations. This disparity highlights different national strategies, contrasting high-impact research (e.g., Canada, Denmark) with high-volume output (e.g., China). Notably, high TLS values (e.g., US, UK) correlate with higher citation impact, emphasizing the role of international collaboration in maximizing research influence.

**Figure 4:**
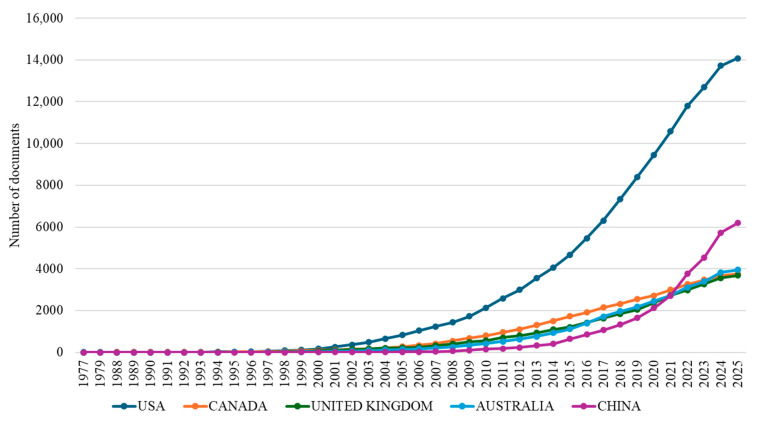
Cumulative scientific production of the five most prolific countries in osteoarthritis assessment and treatment research (1977–2025).

An analysis of temporal trends in scientific output reveals distinct national trajectories in osteoarthritis assessment and treatment research (Figure 4). Examining the cumulative publication output of the five leading nations from 1977 to 2025 highlights significant variations in productivity and periods of rapid growth.

The United States maintained consistent leadership in osteoarthritis research throughout the 1977–2025 period, establishing an early presence and exhibiting sustained growth to reach 14,085 cumulative publications by 2025, reflecting long-term investment. Following the US, the United Kingdom, Canada, and Australia displayed distinct national trajectories but achieved comparable cumulative outputs by 2025. Canada (3760 publications) and the UK (3682 publications) showed largely parallel growth after entering the field in the late 1980s. Australia (3939 publications), despite a later start (1991) and modest initial output, demonstrated significant acceleration post-2010, ultimately surpassing the UK and Canada, correlating with increased national funding. China’s contribution represents the most significant recent shift in the research landscape. Entering the field in 1999, its output remained low until 2010 (149 publications) before undergoing extraordinary expansion, particularly between 2015 and 2025. This exponential growth resulted in 6192 cumulative publications by 2025.

### 3.3. Academic Influence and Impact of Institutions, Journals, and Publications

Bibliometric analysis of 1648 journals publishing on osteoarthritis assessment and treatment (1977–2025) identified the 10 most influential based on citation metrics (Table 2). *Osteoarthritis and Cartilage* dominates, leading significantly in h-index (87), total citations (28,280), and normalized impact via m-index (2.806), alongside substantial publication volume (518 articles). Other key journals exhibit different strengths: *Annals of the Rheumatic Diseases* shows high per-article impact (h-index 73 from 142 articles), the *American Journal of Sports Medicine* demonstrates rapid citation accrual (highest m-index 3.053 despite recent entry), and *BMC Musculoskeletal Disorders* contributes the largest volume (539 articles) despite more moderate citation metrics.

The distribution of bibliometric indicators across different journal types highlights diverse publication profiles. It is evident that specialized journals such as *Osteoarthritis and Cartilage* boast both elevated publication volumes and substantial citation impacts. Conversely, more general rheumatology journals such as *Annals of the Rheumatic Diseases* and *The Journal of Rheumatology* adopt a more selective publication approach, with each article typically garnering considerable influence. Surgical-focused journals, including the *American Journal of Sports Medicine*, the *Journal of Bone and Joint Surgery*, and *Knee Surgery Sports Traumatology Arthroscopy*, form a distinct category, primarily emphasizing operative procedures and functional outcomes in osteoarthritis management.

**Figure 5:**
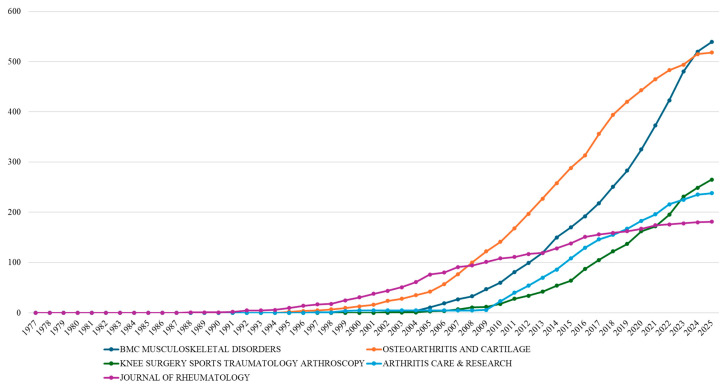
Cumulative publication output of the five most productive journals in osteoarthritis assessment and treatment research (1977–2025).

Analysis of publication output from the most productive journals in osteoarthritis assessment and treatment research reveals distinct temporal patterns that mirror the evolution of the field (Figure 5). The cumulative publication trajectories of these five key journals demonstrate varied engagement over the past few decades. *The Journal of Rheumatology* shows the earliest consistent contribution, starting in 1988, maintaining steady output through the 1990s, and reaching 181 cumulative publications by 2025 through moderate subsequent growth. *Osteoarthritis and Cartilage*, established in 1995 with a specialized focus, exhibited accelerated growth, surpassing *The Journal of Rheumatology*’s output by 2006 and experiencing particularly intensive activity from 2007 to 2020, culminating in 518 articles by 2025 as the second most prolific journal. *BMC Musculoskeletal Disorders*, a more recent entrant from 2003, showed an initial slow start before experiencing extraordinary, nearly ninefold growth between 2010 and 2025 (60 to 539 documents), positioning it as the most prolific journal by 2025. *Arthritis Care & Research* (from 1997) and *Knee Surgery Sports Traumatology Arthroscopy* (from 2002) followed parallel growth patterns, both showing significant increases in output only after 2010 and reaching 238 and 265 cumulative documents, respectively, by 2025.

Among these journals, *BMC Musculoskeletal Disorders* stands out as the only fully open-access journal, as the others operate under hybrid or subscription-based models, offering limited open-access options. The remarkable growth trajectory of *BMC Musculoskeletal Disorders*, from 60 to 539 documents between 2010 and 2025, exemplifies the transformative impact of open-access publishing on osteoarthritis research dissemination. As an open-access journal since its inception in 2003, *BMC Musculoskeletal Disorders* has benefited from increased visibility and accessibility, particularly in emerging research economies. Our analysis reveals that, while this journal shows moderate per-article citation impact (h-index 55, 2.391 m-index), its open-access model has facilitated unprecedented publication volume growth. This pattern contrasts with traditional subscription journals like *Osteoarthritis and Cartilage*, which maintains higher citation metrics (h-index 87, 2.806 m-index) but more modest volume growth.

**Figure 6:**
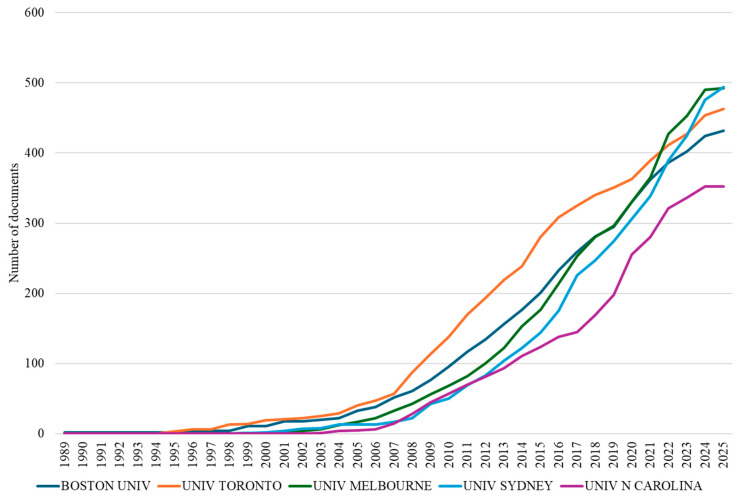
Cumulative publication output of the five most productive institutions in osteoarthritis assessment and treatment research (1989–2025).

The bibliometric analysis of institutional contributions identified 1648 academic and research institutions contributing to osteoarthritis assessment and treatment research (1977–2025). Notably, Australian institutions, particularly the University of Sydney and University of Melbourne, have demonstrated remarkable research capacity development, rising from relative obscurity to achieve leadership positions in publication productivity despite their relatively recent engagement in the field (Figure 6). This Australian prominence likely reflects coordinated national investment in musculoskeletal health research and the development of specialized research centers with extensive international collaborative networks. The institutional analysis reveals that countries with strong international partnerships generally achieve higher citation impacts, underscoring the value of cross-national knowledge exchange in advancing research quality and demonstrating how strategic positioning can facilitate transcontinental knowledge transfer between Western and Asian research communities.

The bibliometric analysis of influential publications in osteoarthritis assessment and treatment research reveals distinctive citation patterns across temporal dimensions. The 1988 publication by Bellamy, which introduced the WOMAC index, is the most cited paper, with 6381 citations, establishing its position as a seminal methodological contribution to the field. Looking at the citation patterns across time, it is fascinating how the landscape has evolved. While classic papers like Bellamy’s work have built impressive citation counts over decades, we are seeing a different pattern with the newer research. Take Hawker’s 2011 paper on pain measurement—it is racking up citations at an astonishing rate of about 218 per year. This is not just a one-off either. When I looked at the top ten most cited papers in the field, I noticed that half of them were published after 2010. This tells me something important is happening: the field is picking up momentum, with knowledge being created and adopted faster than ever before. It seems that researchers are building on each other’s work more quickly, perhaps due to better communication channels or growing interest in osteoarthritis research overall. According to the data, articles published in high-impact journals have a significant citation impact, especially those with an interdisciplinary audience such as *Nature Reviews Disease Primers* (Martel-Pelletier, 2016) and *The Lancet* (Rengister, 2001). The most influential works in this field have been published in specialized journals like *Arthritis Care & Research* (Hawker, 2011) and *The Journal of Rheumatology* (Bellamy, 1988), indicating that targeted distribution to the rheumatology community may maximize citation impact for osteoarthritis assessment research. To further illustrate the citation impact of leading publications in osteoarthritis research, Table 3 presents a comprehensive analysis of citation metrics across influential papers.

### 3.4. Scientific Mapping

**Figure 7:**
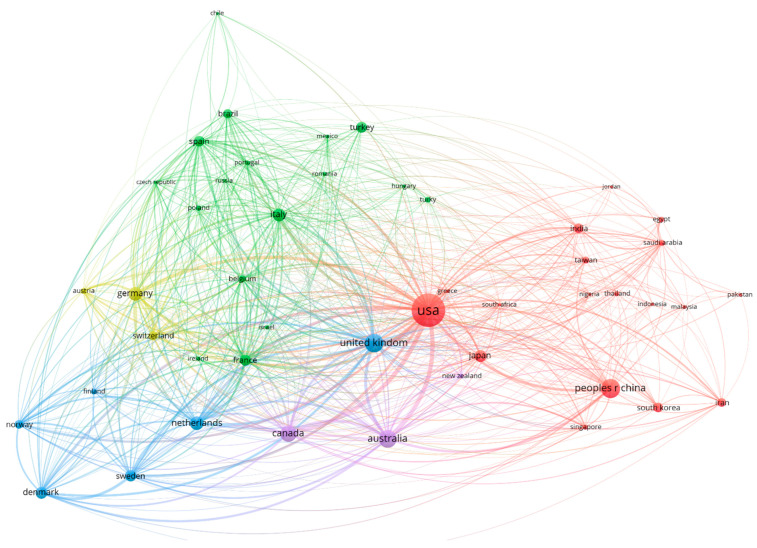
International scientific collaboration network in osteoarthritis assessment and treatment research (1977–2025).

The visualization of the country collaboration network (Figure 7) provides compelling evidence of the complex international research ecosystem in the assessment and treatment of osteoarthritis. Setting a minimum threshold of 30 documents for inclusion resulted in a network of 47 countries, organized into five distinct collaboration clusters that reveal both traditional and emerging partnership patterns. With a high citation volume and a strong emphasis on methodological standardization, the most influential research thematically focuses on evaluation techniques, illness processes, and therapy approaches. Although articles in high-impact interdisciplinary journals also have a considerable citation impact, influential works are primarily found in specialized rheumatology journals. Over the course of the previous three decades, osteoarthritis research has gradually matured, as evidenced by the field’s increasing emphasis on creating standardized evaluation instruments, improving diagnostic methods, and investigating innovative therapy approaches.

As a secondary hub within the blue cluster, the UK maintains 45 collaborative ties and serves as a vital link between the Anglo-American scientific community and continental European research networks. The UK is positioned as a vital bridge in transatlantic knowledge transfer due to its wide-ranging collaboration links with both European research institutes and North American institutions. Australia, symbolized by a big purple node, exhibits exceptional adaptability in collaboration, upholding significant alliances with research communities in both Asia and the West. Australia can act as a transcontinental bridge thanks to its strategic location, which promotes information sharing between historically different research ecosystems.

The green cluster predominantly comprises continental European nations, with Italy, Germany, Spain, and France serving as regional hubs characterized by dense intra-European collaboration. This cluster demonstrates strong internal cohesion, reflecting long-established research partnerships fortified by the European Union. The blue cluster encompasses Nordic countries (Denmark, Sweden, Norway) alongside the Netherlands and Canada, suggesting a collaborative network potentially aligned around shared approaches to healthcare delivery and outcome assessment. With alliances reflecting intricate combinations of historical ties, linguistic similarities, institutional affiliations, and strategic research interests, the collaboration network goes beyond simple geographic proximity. The rise of “bridge countries” such as Australia, which serve to connect historically disparate research communities, points to a changing global research architecture that is more and more defined by transcontinental knowledge networks as opposed to geographically isolated clusters.

**Figure 8:**
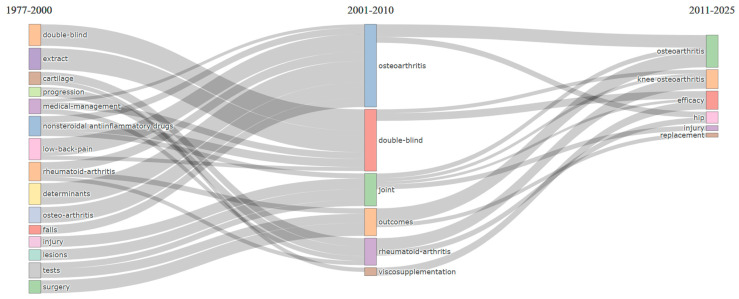
Thematic evolution map of osteoarthritis assessment and treatment research across three time periods (1977–2025).

Analysis of the thematic landscape in osteoarthritis assessment and treatment research reveals a distinct evolution through defined periods, transitioning from compartmentalization towards integrated specialization (Figure 8). The initial foundational period (1977–2000) was characterized by research conducted within discrete thematic silos, often focused on basic differentiations like osteoarthritis versus rheumatoid arthritis, and lacking widespread methodological standardization. A significant shift occurred in the subsequent developmental period (2001–2010), which saw substantial thematic consolidation organized around six primary research domains. A crucial development during this stage was the establishment of standardized outcome measures, a key methodological advance in assessment. This is clearly reflected in the increased frequency and convergence of terms such as “questionnaire”, “validation”, and “womac” within the prominent “outcomes” theme.

Extending this thematic evolution, the most recent expansion period (2011–2025) demonstrates further differentiation into specialized domains. This sub-specialization specifically reflects anatomical sites (e.g., “knee osteoarthritis”, “hip”) and intervention-specific assessment approaches (e.g., “replacement”, “repair”), while maintaining conceptual continuity with established methodological frameworks from the preceding period. This overall evolution, from general ‘tests’ characterized in the earliest period to comprehensive ‘outcome measures’ exhibiting anatomical and intervention specificity in the most recent, underscores the field’s methodological maturation and increasing clinical relevance. A notable development is the thematic bifurcation of ‘outcomes’ research into distinct branches focusing on the evaluation of non-surgical management and surgical interventions, respectively. This represents a sophisticated adaptation of standardized measures for specific clinical applications. This thematic differentiation suggests that future research will continue to refine assessment approaches tailored to specific patient populations and interventions, further enhancing clinical utility while building upon the methodological foundation necessary for evidence-based practice.

**Figure 9:**
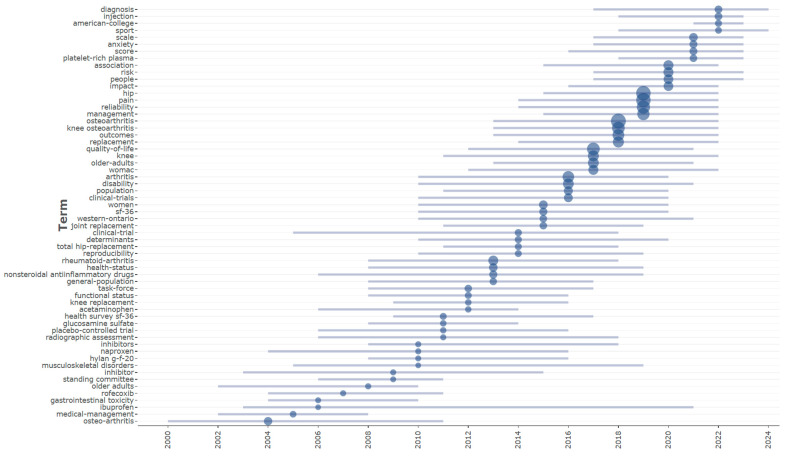
Temporal distribution of trending topics in osteoarthritis assessment and treatment research (2000–2024).

Figure 9 depicts this evolution, displaying the temporal distribution of trending subjects and providing complete insights into the chronological growth of research attention in the domain. The first period (2000–2010) was principally defined by a focus on pharmaceutical interventions and core clinical ideas. Key terms with median years within this decade were “osteo-arthritis” (2004), “medical-management” (2005), and particular pharmaceutical drugs such as “rofecoxib” (2007), “ibuprofen” (2006), and “naproxen” (2010). This perspective reflected the current clinical emphasis on symptom management using anti-inflammatory medicines. The prominence of “gastrointestinal toxicity” (2006) suggests a rising emphasis on the negative effects of these therapies, particularly nonsteroidal anti-inflammatory medications (NSAIDs).

The subsequent intermediate period (2011–2017) marked a substantial shift towards methodological standardization and rigorous outcome assessment. Keywords reflecting these priorities included “health survey sf-36” (2011), “task-force” (2012), and “reproducibility” (2014), signifying increased emphasis on validated assessment instruments and consensus-driven guidelines. This period also marked a clear transition towards the evaluation of surgical interventions, evidenced by the prominence of terms such as “knee replacement” (2012), “total hip-replacement” (2014), and “joint replacement” (2015). The concurrent emergence of “western-ontario” (2015) specifically signals the widespread adoption of the WOMAC index as a standardized tool for assessing osteoarthritis outcomes.

The period from 2018 to 2024 marks a significant evolution toward greater specialization in both assessment methodologies and therapeutic interventions for osteoarthritis. Anatomically specific terminology such as “knee osteoarthritis” (2018) and “hip” (2019) demonstrates the field’s progression toward more precise diagnostic classification and targeted treatment approaches. Concurrently, the literature reflects an expanded focus on the psychosocial dimensions of the condition, evidenced by the increasing prominence of terms like “anxiety” (2021), “impact” (2020), and “people” (2020). This period is further characterized by the emergence of innovative biological therapies, notably “platelet-rich plasma” (2021), signaling a diversification beyond conventional pharmacological and surgical management strategies toward novel biological interventions with potential regenerative properties.

**Figure 10:**
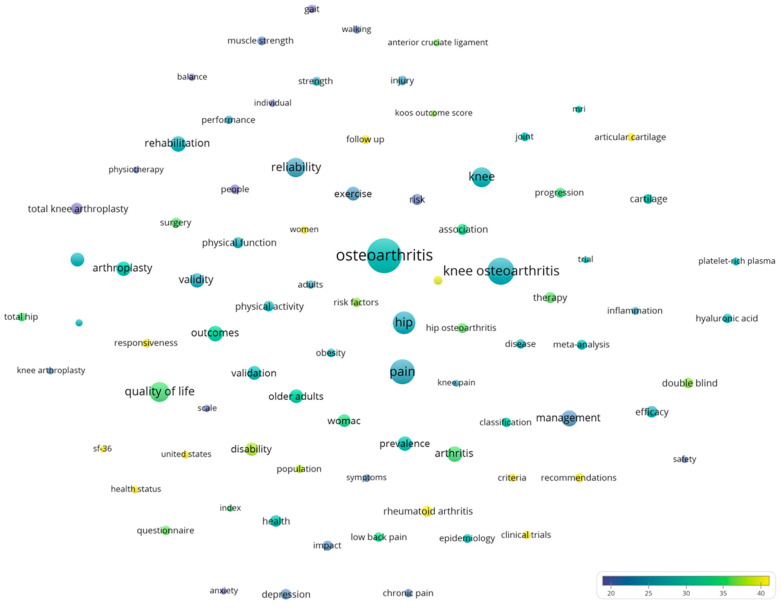
Keyword co-occurrence network in the field of osteoarthritis assessment and treatment research (1975–2025).

The bibliometric examination of the term co-occurrence network reveals a complex intellectual structure behind osteoarthritis evaluation and treatment research, consisting of four distinct yet interconnected topic clusters (Figure 10). This network, made up of 85 high-frequency phrases (with a minimum occurrence criterion of 300), depicts the field’s conceptual architecture and important research objectives from 1977 to 2025. The phrase “Osteoarthritis” (5049 occurrences) acts as the primary node, linking to specialized anatomical domains such as “knee osteoarthritis” (3124 occurrences), “hip” (2298 occurrences), and the important symptom “pain” (2280 occurrences). The spatial distribution of terms inside the network exhibits both strong theme organization and substantive cross-domain links, reflecting the field’s multidisciplinary nature.

Proximal to the central ‘Osteoarthritis’ node, terms related to assessment methodology feature prominently, including “reliability”, “validity”, “outcomes”, and “quality of life”. The strategic positioning of these assessment concepts underscores their fundamental importance in bridging clinical observations with therapeutic evaluations. Standardized assessment instruments, represented by terms such as “womac”, “sf-36”, and “koos outcome score”, are critical tools within this methodological domain.

A key grouping within the network pertains to biological mechanisms and therapeutic targets. Terms such as “cartilage”, “articular cartilage”, “progression”, and “inflammation” form a coherent conceptual area, strategically bridged to clinical assessment domains by “MRI”, highlighting its critical role in translating pathophysiological insights into clinical evaluation.

Surgical and interventional approaches constitute another prominent grouping, with “arthroplasty”, “total knee arthroplasty”, and “surgery” forming a distinct area connected to outcome assessment terms. Terms related to physical function and biomechanics, such as “gait”, “walking”, “muscle strength”, and “balance”, also emerge as important concepts demonstrating the significance of functional assessment. “Rehabilitation” serves as a key bridging term, connecting both surgical interventions and functional assessment concepts.

Citation analysis identifies several high-impact research fronts within the network; “clinical trials”, “cartilage”, and “platelet-rich plasma” demonstrate impact substantially exceeding the field average. Temporal patterns in citation reveal evolving research priorities, with terms like “inflammation” and “platelet-rich plasma” showing more recent average publication years but accelerating citation accumulation.

### 3.5. Funding Sources and Research Support

**Figure 11:**
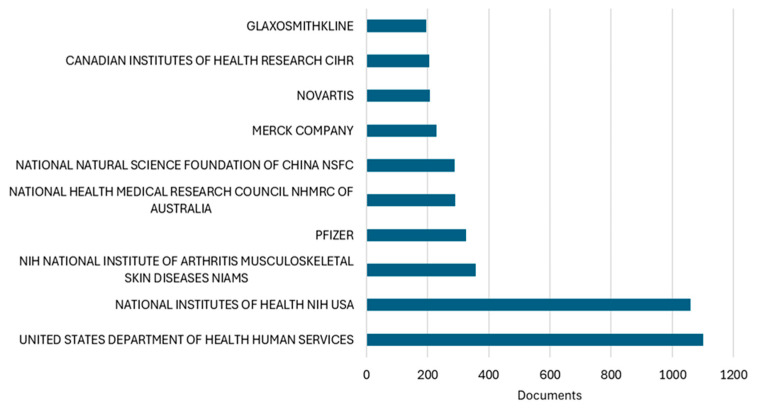
Top funding agencies supporting osteoarthritis assessment and treatment research by publication count.

Analysis of funding acknowledgments revealed diverse financial support patterns underlying osteoarthritis research productivity (Figure 11). Among the analyzed articles with available funding data, the United States Department of Health and Human Services emerged as the predominant funding source, supporting 1100 publications, closely followed by the National Institutes of Health with 1060 publications. The NIH National Institute of Arthritis and Musculoskeletal and Skin Diseases, as a specialized institute, contributed to 358 publications, underscoring targeted federal investment in musculoskeletal research.

The funding landscape demonstrates a notable balance between public and private sector support. While governmental agencies dominate, pharmaceutical companies maintain substantial research investment, with Pfizer supporting 326 publications, followed by Merck (i.e., 230 publications), Novartis (i.e., 208 publications), and GlaxoSmithKline (i.e., 195 publications). This industry engagement aligns with the field’s emphasis on therapeutic development and clinical trials.

International funding patterns reflect the geographic distribution of research productivity identified earlier. The National Health and Medical Research Council of Australia supported 290 publications, corresponding with Australia’s remarkable research trajectory documented in our productivity analysis. Similarly, the National Natural Science Foundation of China funded 288 publications, paralleling China’s exponential growth in research output. The Canadian Institutes of Health Research contributed to 206 publications, consistent with Canada’s high-impact research profile.

## 4. Discussion

The publication output analysis demonstrates a clear pattern of exponential growth, particularly over the past decade, with annual publication volumes increasing from 309 documents in 2010 to 1234 in 2024. This acceleration reflects heightened recognition of osteoarthritis as a global health priority, paralleling the increasing prevalence of this condition in aging populations worldwide.

Citation impact analysis reveals a stable pattern of influence despite the substantial increase in publication volume. The maintenance of citation rates between 3.0 and 4.0 during the mature phase (2005–2019) suggests that the field has achieved methodological consistency while continuing to generate clinically relevant findings. The extraordinary citation performance of seminal assessment tools, particularly Bellamy’s 1988 introduction of the WOMAC index, underscores the foundational importance of standardized outcome measures in advancing osteoarthritis research.

The remarkable ascent of *BMC Musculoskeletal Disorders* as the most prolific journal by 2025, despite its moderate citation impact compared to traditional subscription-based journals, exemplifies how open-access publishing models are reshaping the osteoarthritis research landscape by prioritizing accessibility and global participation over traditional impact metrics.

The geographic distribution of research output reveals both established patterns and significant shifts in scientific leadership within osteoarthritis research. While the United States maintains predominant productivity and impact, the most significant transformation in the international research hierarchy is the rapid emergence of China as the second most prolific contributor. The striking contrast in citation profiles between these two leading nations, with the US demonstrating high impact (39.80 citations per document) compared to China’s lower relative impact (12.70), suggests potential underlying differences in research focus, methodological approaches, or international recognition that warrant further investigation.

On an institutional level, the analysis highlights the remarkable research capacity development at Australian universities; the University of Sydney and University of Melbourne, despite their relatively recent engagement, have established marginal leadership in productivity. This notable Australian prominence in research output likely reflects both coordinated national investment in musculoskeletal health research and the development of specialized research centers actively engaged in international collaborative networks. Indeed, analysis of collaboration patterns reveals that countries with extensive international partnerships generally achieve higher citation impacts, underscoring the significant value of cross-national knowledge exchange in advancing research quality and impact. Australia’s strategic positioning, acting as a “bridge country” connecting Western and Asian research communities, exemplifies how such geographical intermediaries can facilitate valuable transcontinental knowledge transfer and promote methodological standardization.

The funding analysis provides crucial context for understanding the drivers of osteoarthritis research productivity and thematic evolution. The dominance of U.S. federal funding agencies, particularly the specialized NIH National Institute of Arthritis and Musculoskeletal and Skin Diseases institute, correlates with the United States’ sustained leadership in both publication volume and citation impact. The substantial presence of pharmaceutical industry funding (i.e., collectively supporting over 8% of publications) aligns with our thematic analysis showing persistent focus on pharmacological interventions and the evolution toward biologics and novel therapeutics. Notably, the geographic distribution of major funding sources mirrors our productivity findings, with Australian and Chinese funding agencies emerging as significant contributors, reflecting national strategic investments in musculoskeletal health research. The balanced representation of public and private funding suggests a healthy research ecosystem where basic science investigations supported by governmental agencies complement industry-driven translational research. However, the potential influence of funding sources on research directions and publication bias warrants consideration, particularly given the higher representation of pharmaceutical companies in studies focusing on specific therapeutic interventions rather than non-pharmacological management strategies.

Efforts to develop standardized evaluation instruments and measure the effect of osteoarthritis on patient function defined the early phase. With 6381 citations, Bellamy et al.’s 1988 groundbreaking study, which established the WOMAC, is now the most cited article in the area. This patient-reported outcome measure, which offered a disease-specific tool for measuring pain, stiffness, and physical function in osteoarthritis, completely changed the evaluation process [25]. Laupacis and colleagues’ (1993) research established the significant clinical benefits of joint replacement, introduced quality-of-life measurement in surgical outcomes research, and pioneered thorough evaluations of health-related quality of life following total hip replacement. These early investigations created measurements that still have an impact on clinical practice and research techniques, and they served as the basis for further studies on the effectiveness of interventions [26].

During this time, there was a noticeable change in focus toward looking into non-surgical care strategies and possible disease-modifying treatments. The structural effects of glucosamine sulfate were investigated in a seminal randomized controlled experiment by Reginster and colleagues (2001), which introduced the idea of disease modification in the treatment of osteoarthritis and showed potential advantages beyond symptom relief [27]. Important proof that manual physical therapy and supervised exercise can significantly improve functional outcomes for people with osteoarthritis was presented by Deyle and colleagues (2000) [28]. In the Arthritis, Diet, and Activity Promotion Trial (ADAPT), Messier and colleagues (2004) extended this research by showing that moderate exercise and modest weight loss combined to improve functional outcomes and reduce pain in overweight patients with osteoarthritis in their knees [29].

The most recent period (2011–2025) is characterized by further specialization in research foci and increasing methodological approaches, exemplified by several pivotal studies that have shaped the field. Specific advances include an expanded understanding of pain mechanisms: Arendt-Nielsen and colleagues (2010) notably documented the importance of central sensitization, broadening the conceptualization of osteoarthritis pain beyond peripheral joint pathology to include central nervous system changes [30]. Research into non-pharmacological interventions also provided critical evidence, Villareal and colleagues (2011) demonstrated the effectiveness of weight loss and exercise in older adults [31], while the Intensive Diet and Exercise for Arthritis (IDEA) trial by Messier and colleagues (2013) further refined this understanding by documenting both anti-inflammatory effects and biomechanical improvements from these interventions [32].

Comparative effectiveness research emerged as a key priority, providing evidence to inform clinical decision-making. The METEOR trial by Katz and colleagues (2013), for instance, challenged prevailing assumptions about the superiority of surgical approaches for meniscal tears within the context of osteoarthritis [33]. Similarly, a randomized trial by Skou and colleagues (2015) comparing total knee replacement with non-surgical treatment provided crucial evidence for balancing treatment benefits and risks [34]. Furthermore, pain management paradigms underwent substantial evolution, the SPACE trial by Krebs and colleagues (2018) demonstrated that non-opioid medications could provide comparable or even superior pain relief with fewer adverse effects than opioid treatments, fundamentally challenging prevailing management strategies [35].

Several rehabilitative treatments target modifiable risk factors for osteoarthritis and include physical therapy approaches such as manual therapy, neuromuscular training, thermotherapy, electrotherapy (e.g., transcutaneous electrical nerve stimulation, interferential therapy), ultrasound therapy, and shockwave therapy, as well as biomechanical interventions like insole use, footwear modifications, and interactive technologies such as exergaming and virtual reality-based rehabilitation tools.

Manual therapy in osteoarthritis alleviates pain while enhancing joint range of motion and functional capacity by means of both biomechanical adjustments and neurophysiological modulation. This approach involves the mobilization of articular structures and surrounding soft tissues, triggering central analgesic pathways, and providing temporary benefits in patient quality of life [36].

Neuromuscular exercise is essential in managing mild to moderate knee osteoarthritis by enhancing sensorimotor integration, joint stability, and overall functional capacity. Distinct from aerobic or resistance training, neuromuscular exercises target postural control, proprioceptive acuity, and coordinated motor patterns to improve lower limb biomechanics and decrease joint stress. The regimen involves progressively challenging activities such as squats, lunges, step-ups, balance exercises, and functional tasks, all customized to individual tolerance with close monitoring of pain and safety. Outcomes include pain relief, better knee performance, and possible deceleration of disease progression through cartilage preservation. The program is generally well-tolerated, with minimal adverse events, though high-impact exercises like jumping may be less suitable [37].

Thermotherapy is a widely used non-drug approach in osteoarthritis that alleviates pain, enhances joint flexibility, and supports tissue repair by increasing local or systemic temperature. This warming effect improves blood flow, boosts oxygen and nutrient delivery, and facilitates the clearance of metabolic byproducts. Physiologically, heat decreases muscle tension, relaxes connective tissues, and modulates pain receptors to reduce discomfort. On a molecular level, thermotherapy suppresses the NF-κB pathway, lowering pro-inflammatory cytokines like TNF-α, IL-1β, and IL-6, while promoting heat shock proteins such as HSP70 that protect cells and dampen inflammation. It also triggers endorphin release, providing immediate and lasting pain relief. Various modalities include paraffin baths, infrared, deep heat (diathermy, ultrasound), spa therapies, and heat packs, each improving symptoms and function. These treatments should be tailored individually and are generally safe, forming a key adjunct in OA management [38].

Electrotherapy represents a widely adopted, non-invasive, and cost-efficient intervention for symptom control in osteoarthritis, particularly affecting the knee. This technique delivers electrical impulses via skin-mounted electrodes to alleviate pain and enhance joint functionality. Its mechanism involves altering nociceptive transmission according to the gate control theory and promoting endogenous endorphin secretion, resulting in analgesia. Additionally, electrotherapy improves regional circulation, diminishes inflammatory responses, and facilitates muscle activation surrounding the involved joints, thereby supporting better mobility and overall patient quality of life [39].

The main types of electrotherapies are Transcutaneous Electrical Nerve Stimulation (TENS), Interferential Current Therapy (IFC), neuromuscular electrical stimulation, and pulsed electrical stimulation. Interferential Current Therapy has shown the most consistent and effective pain relief due to its deeper tissue penetration and ability to overcome skin resistance using medium-frequency currents. High-frequency TENS is also commonly employed, providing analgesia by blocking pain signal transmission [40].

Overall, electrotherapy provides various physiological advantages, including pain control, inflammation reduction, improved blood flow, and muscle activation, with minimal adverse effects reported. Although additional studies are necessary to clarify its long-term efficacy, ET remains a useful complementary approach in osteoarthritis treatment, especially during early to moderate stages when surgical intervention is not yet indicated [39].

Therapeutic ultrasound is a commonly employed physical modality aimed at alleviating pain and facilitating soft tissue repair in patients with knee osteoarthritis. It exerts two primary actions: thermal and non-thermal. Continuous ultrasound generates heat within tissues, enhancing circulation, metabolic activity, and tissue pliability, thereby contributing to pain relief. In contrast, pulsed ultrasound influences cellular membranes, stimulates protein production, and activates immunological processes that support tissue regeneration. Treatment parameters such as frequency and intensity are adjusted according to therapeutic objectives, with continuous ultrasound targeting thermal effects and pulsed ultrasound delivering non-thermal benefits. Current evidence suggests that ultrasound therapy can modestly decrease pain and improve functional outcomes in knee osteoarthritis without causing significant adverse effects. Despite the generally low quality of evidence, ultrasound remains a safe, non-invasive adjunctive treatment that may also help minimize reliance on pharmacological interventions [41].

Extracorporeal shockwave therapy represents a non-invasive treatment approach with increasing promise in osteoarthritis management, effectively reducing pain and enhancing joint function. This method applies high-energy shockwaves generated by electrohydraulic, electromagnetic, or piezoelectric sources directly to affected tissues. The therapy promotes cartilage preservation by stimulating chondrocyte activity, decreasing cartilage damage and programmed cell death, and improving the health of subchondral bone. Additionally, it supports tissue regeneration, encourages new blood vessel growth, and modulates inflammatory processes, all contributing to pain reduction and improved joint mobility. While optimal treatment protocols are still being defined, extracorporeal shockwave therapy offers a safe and effective option for symptom relief and slowing the progression of osteoarthritis [42].

Biomechanical strategies in osteoarthritis management aim to modify joint loading and alignment, thereby alleviating pain and enhancing function by targeting issues such as joint malalignment, muscle weakness, and abnormal movement patterns. These interventions encompass the use of braces, orthotic shoe insoles or wedges, gait retraining, and assistive devices including canes. Insoles and footwear adjustments, like medial or lateral wedges, function to alter the center of pressure during gait, diminishing the external knee adduction moment and consequently reducing stress on affected joint compartments, which may lessen symptoms. Knee bracing, particularly valgus (unloader) braces, exerts corrective forces to improve joint stability and alignment, while gait retraining modifies walking mechanics to decrease deleterious joint loading. Proper use of canes also contributes to joint load reduction during ambulation. These methods are typically non-invasive, cost-effective, and present minimal adverse effects, though their success largely depends on individualized fitting and patient compliance. Despite promising biomechanical improvements and symptom relief, the evidence for sustained clinical benefits over the long term remains inconsistent, emphasizing the importance of tailored therapeutic approaches [43].

Innovative interactive approaches such as exergaming and virtual reality-based rehabilitation offer engaging, motivating environments for patients with osteoarthritis, facilitating improvements in pain management, muscle strength, and overall functional capacity. Virtual reality systems, whether immersive or non-immersive, provide visual biofeedback alongside adjustable difficulty levels, aiding in movement correction and enhancing joint function and postural balance. These technologies improve accessibility and promote greater patient adherence compared to conventional therapeutic methods. Evidence indicates that virtual reality exercise significantly reduces pain and strengthens muscles while enhancing overall osteoarthritis outcome measures, although its impact on joint stiffness and physical functionality remains less definitive. Given the limited quantity of current research, further studies are necessary to better establish these effects [44,45].

To date, no pharmacological agent has been shown to completely stop or reverse structural changes in knee osteoarthritis. Management strategies encompass non-pharmacological strategies like weight loss, exercise, and lifestyle modification; medications such as paracetamol, nonsteroidal anti-inflammatory drugs, opioids, and intra-articular injections; and surgery, typically reserved for late-stage disease. Recent developments in intra-articular (IA) therapies for knee osteoarthritis reflect growing interest in both traditional and regenerative options. In clinical practice, the most commonly used IA injections include corticosteroids, hyaluronic acid (HA), platelet-rich plasma (PRP), stem cells, and prolotherapy. IA delivery enhances local therapeutic efficacy while limiting systemic exposure and off-target effects by directly targeting the joint space [46].

Interest in dextrose prolotherapy (DPT) as a regenerative IA treatment for knee osteoarthritis has increased due to its affordability and accessibility relative to conventional therapies. Although earlier research was limited and lacked uniform protocols, recent evidence highlights its safety and promising clinical benefits [47]. Meta-analyses indicate that DPT significantly alleviates pain and stiffness while enhancing function, as reflected by improved WOMAC scores, with effects more pronounced in elderly or higher BMI patients. The mechanism involves controlled inflammation promoting tissue repair through fibroblast activation and ligament strengthening. Compared to placebo, corticosteroids, HA, and PRP, DPT has yielded comparable or superior outcomes [48]. Furthermore, a systematic review including over 800 patients supports DPT’s efficacy in alleviating pain, stiffness, and functional impairment, as measured by WOMAC. While DPT’s pain relief is similar to PRP at six months, it is somewhat less effective in reducing stiffness. Proposed mechanisms include inflammation-driven repair, fibroblast proliferation, chondrogenesis, and direct analgesic effects. Despite moderate-quality evidence owing to methodological heterogeneity (e.g., dextrose concentration, injection frequency), DPT is considered safe, with no significant adverse events reported [49].

Since 2008, the use of PRP as a treatment modality for knee osteoarthritis has seen a significant rise in attention. This surge is largely due to predominantly positive findings from both clinical and preclinical studies. Multiple meta-analyses evaluating PRP’s efficacy compared to other interventions consistently demonstrate superior pain relief and functional gains at various follow-up intervals. Importantly, PRP has shown a favorable safety profile without added risks, positioning it as a viable conservative therapeutic option [50]. Mechanistically, once activated, PRP forms a tridimensional gel-like scaffold that traps growth factors and biomolecules critical for cellular proliferation, differentiation, and migration within regenerating tissue. Comparative studies assessing PRP against standard injectables such as HA, corticosteroids, placebo, mesenchymal stem cells, and PRP variants (e.g., leukocyte-poor or leukocyte-rich PRP) reveal that PRP generally achieves greater symptomatic improvement over 6 to 12 months [51].

Guidelines from the European Society of Sports Traumatology, Knee Surgery and Arthroscopy—International Cartilage Repair Society consensus recommend PRP injections as appropriate for patients up to 80 years old with Kellgren–Lawrence grades 0 to III knee osteoarthritis who have not responded to prior conservative treatments. However, PRP is not advised as an initial therapy nor for advanced osteoarthritis (Kellgren–Lawrence grade IV) [52].

Combination therapies in knee osteoarthritis show promising advantages over single-agent treatments. Notably, the intra-articular use of corticosteroids combined with hyaluronic acid appears to provide superior symptom relief compared to corticosteroids alone, although confirmation through larger clinical trials remains necessary. In parallel, the dual application of platelet-rich plasma and hyaluronic acid has emerged as an innovative strategy, especially effective in managing grade II–III knee osteoarthritis by synergistically enhancing pain reduction and functional improvement beyond what either treatment achieves individually [46,53]. Additionally, combining intra- and extra-articular DPT offers a complementary approach. While corticosteroids produce more immediate pain relief, prolotherapy’s longer-lasting effects become evident at mid-term follow-up, suggesting that prolotherapy, particularly when combined with other injection techniques, may offer sustained benefits superior to corticosteroids alone [54].

Collectively, these studies and the research trends they represent illustrate the increased specialization, methodological sophistication, and direct clinical relevance that characterized osteoarthritis research during this recent period. They highlight a move towards a more nuanced understanding of the disease, evidence-based comparisons of treatment modalities, and refined management approaches that integrate complex aspects of pain and function.

To better contextualize the contributions of the present study within the landscape of osteoarthritis bibliometric research, Table 4 provides a summary of recent bibliometric analyses in this field. This comparative overview highlights essential features such as database sources, study durations, dataset sizes, analytical tools, and thematic focus areas. Our study distinguishes itself by delivering a more comprehensive and clinically oriented perspective, spanning a longer timeframe and utilizing advanced computational techniques for data standardization. This expanded scope and methodological innovation position our work as a valuable addition to existing literature, addressing gaps present in prior analyses that tend to focus on narrower OA subdomains or shorter study periods.

Our bibliometric analysis of osteoarthritis diagnosis and management stands out among current studies in the field due to its distinct approach, methodological novelty, and broad thematic integration. While many bibliometric analyses within osteoarthritis research concentrate on specific and narrow subdomains, the present study offers a broader and more integrative perspective by focusing on the clinical aspects of osteoarthritis. This includes an in-depth examination of diagnostic and therapeutic assessment tools such as WOMAC and KOOS, areas that have received comparatively less attention in prior analyses.

A key distinguishing feature is the temporal depth of the analysis. Covering the period from 1977 to 2025, this study spans 49 years, representing the longest timeframe observed in the reviewed literature containing bibliometric analyses targeting osteoarthritis research. In contrast, previous analyses typically examine research output across 10 to 22 years. This extended timeframe enables the identification of long-term research trajectories and paradigm shifts, and contextualizes the exponential growth in osteoarthritis-related publications within a robust historical framework.

Another notable aspect is the development of an advanced Boolean-based search strategy. Structured around three major thematic dimensions (i.e., clinical assessment and therapeutic interventions), this algorithm incorporates over 40 individual terms, one of the largest in osteoarthritis bibliometric research. The use of nested Boolean operators (“AND”, “OR”) allows for highly specific yet inclusive retrieval of relevant literature. This rigorous strategy yielded a robust and highly relevant dataset comprising 12,661 articles. To the best of our knowledge, this represents the largest and most refined dataset identified in any bibliometric study within the field of osteoarthritis.

A novel computational element was introduced through the use of Python 3.12.3-based TF-IDF vectorization, semantic clustering, and cosine similarity for automated keyword standardization. This machine learning approach introduces a level of semantic rigor and reproducibility that significantly enhances the quality of co-word analysis and thematic clustering. Furthermore, the analysis of international collaboration networks, including the identification of “bridge countries” such as Australia that link Western and Asian research hubs, provides a layer of geopolitical insight scarcely addressed in existing literature.

Regarding tools and platforms, this study is among the few to synergistically combine VOSviewer, Bibliometrix, and Excel. Despite certain limitations shared with previous studies (i.e., reliance primarily on a single database and English-only literature), this work is distinct in its breadth, depth, and technical innovation. The integration of a machine learning-powered thesaurus generator represents one of the most methodologically advanced and thematically relevant bibliometric contributions to the field of osteoarthritis to date.

Despite being thorough, this bibliometric analysis has a few drawbacks. First, although it offers consistency, depending only on one database (i.e., Web of Science Core Collection) may leave out pertinent papers that are only indexed in other databases. While our reliance on Web of Science Core Collection as the sole data source ensures methodological consistency and data quality, we acknowledge that this approach may not capture all osteoarthritis literature indexed exclusively in other databases. However, recent comprehensive database comparison studies demonstrate that approximately 99% of Web of Science journals are also covered by other major databases, suggesting minimal loss of high-quality literature. Furthermore, Singh et al. showed that combining multiple databases introduces significant methodological complications, including variations in research output volumes and rankings that can exceed 10% for the same countries across different databases. Our single-database approach was therefore chosen to maintain analytical consistency and avoid systematic biases that could arise from database-specific indexing protocols and coverage patterns, particularly important for our five-decade longitudinal analysis [21].

Additionally, our restriction to English-language publications, while methodologically necessary for keyword and thematic analyses, may underrepresent research contributions from non-English speaking regions. This limitation could potentially exclude important regional research traditions, local treatment approaches, or population-specific findings published in languages such as Chinese, Japanese, Spanish, German, etc. The exclusion is particularly relevant given that countries like China and Japan appear in our top contributors but show lower citation impacts, which may partially reflect the exclusion of their non-English publications. Future analyses incorporating multilingual approaches or regional databases could provide a more globally inclusive perspective on osteoarthritis research trends.

Second, the research only includes official academic publications, not targeting additional channels for disseminating knowledge, such as policy documents, clinical recommendations, and instructional materials that could have a big impact on practice.

Despite being standardized, citation metrics could not accurately reflect the caliber of research or its practical applications. While therapeutically transformational research may produce modest citation counts despite significant practical value, high-impact methodological studies may gather citations without necessarily translating into improved patient outcomes.

Alternative impact metrics, multi-database techniques, and supplementary qualitative evaluations of study content could also be used in future bibliometric studies to overcome these constraints. Further insights into the translational trajectory from research to clinical implementation may be obtained by conducting further analyses that look at funding trends, clinical trial registration, and patent filings.

The identified research trends suggest several promising directions for future investigation. The emerging focus on personalized assessment approaches tailored to specific patient subpopulations and anatomical locations indicates potential for increasingly precise intervention targeting. The growing attention to the psychosocial dimensions of osteoarthritis, including anxiety and depression, suggests opportunities for integrated assessment frameworks addressing both physical and psychological aspects of this condition.

## 5. Conclusions and Prospects

This thorough bibliometric review of research on osteoarthritis assessment and therapy from 1977 to 2025 shows that the field has grown exponentially throughout four distinct phases, indicating the growing awareness of osteoarthritis as a serious worldwide health concern. WOMAC and KOOS represent essential tools for assessing symptoms, function, and quality of life in knee osteoarthritis, guiding treatment decisions.

Although there are large differences in the impact of citations (39.80 vs. 12.70 citations per document), China’s rapid growth signifies a major geopolitical shift, and the United States continues to lead the world in some important categories. Australian institutions are prime examples of how focused national investment may quickly improve research stature, having risen from relative obscurity to hold prominent positions. Collaboration network analysis reveals a sophisticated ecosystem structured around five distinct clusters with strategically positioned “bridge countries” facilitating knowledge transfer between historically disparate research communities.

The funding landscape analysis reveals a strong correlation between sustained governmental investment and research leadership and citation impact. The balanced representation of public and private funding sources, with pharmaceutical companies supporting approximately 8% of publications, reflects a healthy research ecosystem where basic science and translational research complement each other. This finding reinforces the significance of employing a variety of funding strategies to ensure the sustained advancement of research in osteoarthritis science and to encourage innovation in this field.

However, there are still several important research gaps, such as the relative underrepresentation of non-Western viewpoints outside of China and Japan and the poorly understood connection between publishing metrics and actual therapeutic impact. Future directions emerging from this analysis include the development of more sophisticated clinimetric and psychometric instruments tailored to specific patient phenotypes and anatomical sites, addressing the need for increasingly personalized assessment approaches.

Integration of multidimensional psychometric measures capturing both physical and psychological dimensions of osteoarthritis represents another promising frontier, as does the standardized evaluation of emerging biological interventions. As global populations age and osteoarthritis prevalence rises, this bibliometric analysis provides not merely a retrospective examination of research trends, but a foundation for strategically advancing osteoarthritis science toward increasingly impactful clinical applications, where methodologically robust assessment approaches will continue to serve as the cornerstone for evidence-based practice and precision medicine approaches to this complex condition.

## Figures and Tables

**Table 1:** Bibliometric indicators of national scientific contribution to osteoarthritis assessment and treatment research (1977–2025).

Country	Documents	Citations	Average Citations/Document	TLS
USA	3094	123,151	39.80	2000
China	1080	13,717	12.70	365
United Kingdom	1045	39,987	38.27	1334
Australia	941	36,737	39.04	1037
Canada	841	47,007	55.89	868
Netherlands	604	19,485	32.26	737
Germany	586	20,668	35.27	826
Italy	518	16,274	31.42	646
Denmark	474	20,001	42.20	589
Japan	471	7740	16.43	159

TLS, total link strength.

**Table 2:** Impact of the most influential journals in osteoarthritis research and publication.

Source	h_Index	g_Index	m_Index	Total Citations	Publications	Publication Start
OSTEOARTHRITIS AND CARTILAGE	87	135	2.806	28,280	518	1995
ANNALS OF THE RHEUMATIC DISEASES	73	123	2.028	15,415	142	1990
JOURNAL OF RHEUMATOLOGY	61	129	1.605	17,186	181	1988
AMERICAN JOURNAL OF SPORTS MEDICINE	58	96	3.053	9994	176	2007
BMC MUSCULOSKELETAL DISORDERS	55	87	2.391	12,351	539	2003
ARTHRITIS CARE & RESEARCH	54	107	1.862	13,155	238	1997
JOURNAL OF BONE AND JOINT SURGERY-AMERICAN VOLUME	50	92	1.515	8671	106	1993
ARTHRITIS & RHEUMATISM-ARTHRITIS CARE & RESEARCH	47	77	1.88	6404	77	2001
KNEE SURGERY SPORTS TRAUMATOLOGY ARTHROSCOPY	45	78	1.875	8116	265	2002
ARTHRITIS AND RHEUMATISM	43	55	1.162	8101	55	1989

h-index, Hirsch index; g-index, Egghe’s g-index; m-index, m-quotient.

**Table 3:** Citation metrics and normalized impact of leading publications in osteoarthritis research.

Paper/Source	TC	TC/Year	Normalized TC	DOI
BELLAMY N, 1988, J RHEUMATOL	6381	167.92	1.00	NA
HAWKER GA, 2011, ARTHRITIS CARE RES-A	3265	217.67	50.15	10.1002/acr.20543
Pedersen Bk, 2015, Scand J Med Sci Sports	1419	129.00	35.85	10.1111/sms.12581
Neogi T, 2013, Osteoarthritis Cartilage	1083	83.31	24.24	10.1016/j.joca.2013.03.018
Martel-Pelletier J, 2016, Nat Rev Dis Primers	1064	106.40	31.20	10.1038/nrdp.2016.72
Lohmander Ls, 2004, Arthritis Rheum	1060	48.18	9.67	10.1002/art.20589
Collins Nj, 2011, Arthritis Care Res	900	60.00	13.83	10.1002/acr.20632
Reginster Jy, 2001, Lancet	878	35.12	6.34	10.1016/S0140-6736(00)03610-2
Kohn Md, 2016, Clin Orthop Rel Res	850	85.00	24.93	10.1007/s11999-016-4732-4
Apovian Cm, 2015, J Clin Endocrinol Metab	809	73.55	20.44	10.1210/jc.2014-3415

TC, total citations; NA, not applicable.

**Table 4:** Comparative table of bibliometric analyses targeting osteoarthritis research.

Study Title/ First Author	Study Objectives	Database	Time Span	No. of Papers	Tools Used	Analytical Depth	Limitations	Ref.
Foot Osteoarthritis Research: A Bibliometric Analysis/Menz A.	Map non-surgical foot OA research trends and gaps	Scopus	Inception–Dec 2023	121	Biblioshiny^®^ (R/bibliometrix), Excel, Covidence	Citations, authors, institutions, countries, journals; categorization by UKCRC research type; analysis of funding sources and collaboration patterns	Limited to English-language, non-surgical studies; excluded case reports, editorials, and non-indexed works; possible funding under-reporting; keyword normalization not discussed; single-author classification for research type and funding; lacks societal impact assessment	[18]
Bibliometric Analysis of Publications in Clinical Trials on Knee Osteoarthritis Between 2001 and 2022/Gu J. Y.	Map and analyze clinical trials on knee OA treatments	Web of Science Core Collection	Jan 2001–May 2022	1972	Excel, GraphPad Prism, CiteSpace, VOSviewer	Trends in publications, countries, authors, institutions, journals; keyword clustering; citation bursts; network and cluster analyses; treatment types identified by co-occurrence and burst keyword detection	Only Web of Science searched; possible duplicate or missed authors/keywords due to name variations; MeSH used externally to compensate for database limitations	[55]
A Bibliometric and Knowledge Map Analysis of Osteoarthritis Signaling Pathways from 2012 to 2022/Li B.	To map and visualize research on OA signaling pathways, identify key contributors, hotspots, and trends	Web of Science	2012–2022	4894	CiteSpace, VOSviewer, Scimago Graphica, Excel	Analysis of countries, institutions, authors, cited references, and keywords, highlighting key pathways and exosomes	Limited to WoS; potential inclusion of irrelevant articles due to large dataset and lack of manual filtering	[19]
Bibliometric analysis of research on osteoarthritis and extracellular vesicles: Trends and frontiers/Ding Y.	To assess trends, hotspots, and contributors in EV-related OA research and guide future directions	Web of Science Core Collection	2003–2022	354	CiteSpace, VOSviewer, R package “bibliometrix”, Origin, Draw Venn outline online website	Evaluated countries, institutions, authors, journals, co-citations, keywords; identified core topics such as diagnosis, drug delivery, therapy, and cartilage repair	Limited to one database and English-language publications; 2023 data not fully captured; exclusion of non-article materials	[20]
Research progress and hot spot analysis related to oxidative stress and osteoarthritis: a bibliometric analysis /Gu J.Y.	To conduct a bibliometric analysis of trends, key authors, countries, institutions, journals, and research topics in oxidative stress and osteoarthritis	Web of Science	1998–2022	1412	VOSviewer, Citespace, Excel	Identification of leading authors, countries, institutions, influential journals, keyword co-occurrence, and 9 keyword clusters representing research hotspots	Limited international collaboration; database and language limitation (exclusive use of only one database targeting only English-written papers)	[56]
Bibliometric analysis of research trends in stem cell therapy for knee osteoarthritis over the period 2001–2021/Chen R.	To analyze global research trends, key contributors, hotspots, and thematic evolution in stem cell therapy for knee OA	Web of Science Core Collection	2001–2021	1345	VOSviewer, CiteSpace, Excel	Analysis of publication output, authorship, institutions, countries, keyword co-occurrence, citation bursts, research hotspots, and thematic trends	Language limited to English; only original articles included; data snapshot on one day may omit later updates; word duplication in keyword counts	[57]
Mapping knowledge landscapes and emerging trends of the links between osteoarthritis and osteoporosis: A bibliometric analysis /Wan X.	To explore global research trends, collaboration networks, and key hotspots in the OA–OP research domain	Web of Science Core Collection (SCI-Expanded)	1998–2021	1078	CiteSpace, VOSviewer, Excel, bibliometric.com	Detailed analysis of authorship, co-citations, institutional and national collaboration, keyword co-occurrence	Limited to Web of Science Core Collection and English-only articles; possible exclusion of recent updates; non-WoS studies omitted	[58]
Mapping the knowledge landscape: A bibliometric analysis of exosome research in osteoarthritis (2004–2023)/Xu H.	To analyze global trends, research hotspots, and emerging directions in exosome-related OA research	Web of Science Core Collection (SCI-Expanded)	2004–2023	456	VOSviewer, CiteSpace, Excel, Scimago Graphica	Author, institution, keyword, co-citation, trend, and burst analysis	Data limited to Web of Science Core Collection; non-English and non-WoS literature excluded; clinical trials underrepresented	[59]
Bibliometric and visualization analysis of macrophages associated with osteoarthritis from 1991 to 2021/ Yang Z.	To map research trends, hotspots, and knowledge structure in macrophage-related OA studies	Web of Science Core Collection	1991–2021	1481	CiteSpace, VOSviewer, bibliometrix (R), Excel, Origin, GraphPad Prism 8	Co-citation, keyword clusters, dual-map overlay, country/institution/author/journal networks	Limited to Web of Science Core Collection and English-language studies; excluded other databases; no keyword timeline; possible bias in data selection	[60]
Mapping the Knowledge Landscape of and Emerging Future Trends in Stem Cell Therapy for Osteoarthritis: A Bibliometric Analysis of the Literature From 1998 to 2024/ Meng Z.	To analyze and visualize trends, hot topics, and future directions in OA stem cell therapy	Web of Science Core Collection	1998–2024	2341	CiteSpace, VOSviewer	Keyword co-occurrence, collaboration networks, citation analysis, trend prediction	Limited to Web of Science Core Collection; only articles and reviews; possible keyword repetition bias; fixed-date search may exclude recent updates	[61]
A bibliometrics analysis and visualization of osteoimmunology on osteoarthritis studies/Lu B.	To analyze global research trends and hotspots in OA-related osteoimmunology	Web of Science	1991–2020	1004	SPSS version 24, GraphPad Prism, CiteSpace, VOSviewer	Citation analysis, keyword co-occurrence, country/institution mapping	Only English Web of Science articles; excluded 2021; possible search bias; underrepresents recent and non-English research	[62]

UKCRC, United Kingdom Clinical Research Collaboration; OA, osteoarthritis; OP, osteoporosis.

## Data Availability

The raw data supporting the conclusions of this article will be made available by the authors on request.

## Abbreviations

The following abbreviations are used in this manuscript:

WOMAC	Western Ontario and McMaster Universities Osteoarthritis Index
KOOS	Knee Injury and Osteoarthritis Outcome Score
TLS	Total Link Strength
TF-IDF	Term Frequency-Inverse Document Frequency
IA	Intra-articular
HA	Hyaluronic Acid
PRP	Platelet-Rich Plasma
DPT	Dextrose Prolotherapy
TENS	Transcutaneous Electrical Nerve Stimulation
IFC	Interferential Current Therapy
